# Hematopoietic Stem Cells in Neural-crest Derived Bone Marrow

**DOI:** 10.1038/srep36411

**Published:** 2016-12-21

**Authors:** Nan Jiang, Mo Chen, Guodong Yang, Lusai Xiang, Ling He, Thomas K. Hei, Gregory Chotkowski, Dennis P. Tarnow, Myron Finkel, Lei Ding, Yanheng Zhou, Jeremy J. Mao

**Affiliations:** 1 Department of Orthodontics, Peking University School and Hospital of Stomatology 22 Zhongguancun Avenue South, Haidian District, Beijing 100081, P.R. China; 2 Columbia University Medical Center, Center for Craniofacial Regeneration (CCR) 630 W. 168 St., New York, NY 10032, USA; 3 Central Laboratory, Peking University School and Hospital of Stomatology, 22 Zhongguancun Avenue South, Haidian District, Beijing 100081, China; 4 Guanghua School of Stomatology, Hospital of Stomatology, Sun Yat-sen University 56 Lingyuanxi Road, Guangzhou 510055, China; 5 Department of Radiation Oncology, College of Physician and Surgeons Columbia University Medical Center, 630 W. 168 St., New York, NY 10032, USA; 6 Division of Periodontics, College of Dental Medicine, Columbia University Medical Center 630 W. 168 St., New York, NY 10032, USA; 7 Departments of Microbiology & Immunology and Rehabilitation & Regenerative Medicine 630 W. 168 St., New York, NY 10032, USA

## Abstract

Hematopoietic stem cells (HSCs) in the endosteum of mesoderm-derived appendicular bones have been extensively studied. Neural crest-derived bones differ from appendicular bones in developmental origin, mode of bone formation and pathological bone resorption. Whether neural crest-derived bones harbor HSCs is elusive. Here, we discovered HSC-like cells in postnatal murine mandible, and benchmarked them with donor-matched, mesoderm-derived femur/tibia HSCs, including clonogenic assay and long-term culture. Mandibular CD34 negative, LSK cells proliferated similarly to appendicular HSCs, and differentiated into all hematopoietic lineages. Mandibular HSCs showed a consistent deficiency in lymphoid differentiation, including significantly fewer CD229 + fractions, PreProB, ProB, PreB and B220 + slgM cells. Remarkably, mandibular HSCs reconstituted irradiated hematopoietic bone marrow *in vivo,* just as appendicular HSCs. Genomic profiling of osteoblasts from mandibular and femur/tibia bone marrow revealed deficiencies in several HSC niche regulators among mandibular osteoblasts including Cxcl12. Neural crest derived bone harbors HSCs that function similarly to appendicular HSCs but are deficient in the lymphoid lineage. Thus, lymphoid deficiency of mandibular HSCs may be accounted by putative niche regulating genes. HSCs in craniofacial bones have functional implications in homeostasis, osteoclastogenesis, immune functions, tumor metastasis and infections such as osteonecrosis of the jaw.

Hematopoietic stem cells (HSCs) undergo self-renewal and differentiate into all blood lineages. HSCs have been studied extensively in bone marrow of mesoderm derived appendicular and axial skeletons with a demonstrated capacity to restore irradiated bone marrow by single clonal progenies^1^. HSCs reside in bone marrow niche that not only provides stromal support, but also signaling functions^2^. Remarkable progress has been made in the characterization of appendicular HSCs and their niche^3^,^4^, contributing to clinical applications of bone marrow transplantation^5^,^6^. Different from mesoderm-derived appendicular bones, facial bones derive from neural crest cells that migrate from the neural tube to form branchial arches^7^. Neural crest cells initiate from the cells at the border between neural and non-neural ectoderm^8^,^9^. A subset of neural crest cells migrate to the presumptive face and form multiple craniofacial tissues including the maxilla and mandible, dental mesenchyme, Merckel’s cartilage and the temporomandibular joint^10^,^11^. The mouse embryonic head at the gestation stage of E12.5 is a site of robust hematopoiesis with long-term, self-renewable HSCs^12^. However, whether neural crest-derived craniofacial skeleton harbors HSCs postnatally is elusive. Lack of knowledge of postnatal hematopoiesis in craniofacial skeleton impairs our understanding of not only homeostasis including osteoclastogenesis, but also a broad range of pathological conditions including osteonecrosis, primary or metastasized facial bone malignancies and infections such as osteomyelitis^13^.

HSCs are maintained and regulated in the microenvironments called niches in which preserve the properties including cells and signal molecules. Cxcl12 (chemokine C-X-C motif) ligand, expressed by stromal niche cell populations, is a key chemokine that regulate HSC functions^14^,^15^,^16^. Conditional deletion of Cxcl12 from perivascular stromal cells affected HSCs proliferation, self-renew and trafficking and depleted certain restricted progenitors^17^. Cxcl12 is also required for normal B- and T cell development^18^. HSCs interact with various niche cells in bone marrow including perivascular stromal cells, mesenchymal stromal cells, endothelial cells, osteoblasts, macrophage, adipocytes and sympathetic neurons in ways that require further understanding^19^,^20^,^21^. Osteoblasts have received robust attention for HSCs in bone marrow. Transplanted HSCs preferentially home to trabecular bone rather than the diaphysis, as osteoblasts in trabecular bone express a unique set of homing factors^22^. Niche cells not only regulate HSC maintenance and differentiation, but also exert complex signals. Conditional deletion of *Cxcl12* from osteoblasts (Col2.3-Cre) depletes early lymphoid progenitors but not HSCs and cellularity in bone marrow^23^. Compared to iliac crest bone marrow stromal cells, facial bones including the maxilla and mandible have rich vasculature, with bone marrow stromal cells proliferating at more rapid rates, and formed more ectopic bone *in vivo*^24^, suggesting that neural crest-derived stromal cells in facial bones may preserve different stromal microenvironment for putative HSCs. In this study, we identified hematopoietic stem cells in the mandible and benchmarked mandibular HSCs with donor-matched appendicular HSCs isolated from donor-matched femur/tibia.

## Results

### CD34ˉLSK cells from neural crest-derived mandibular bone

**Figure 1:**
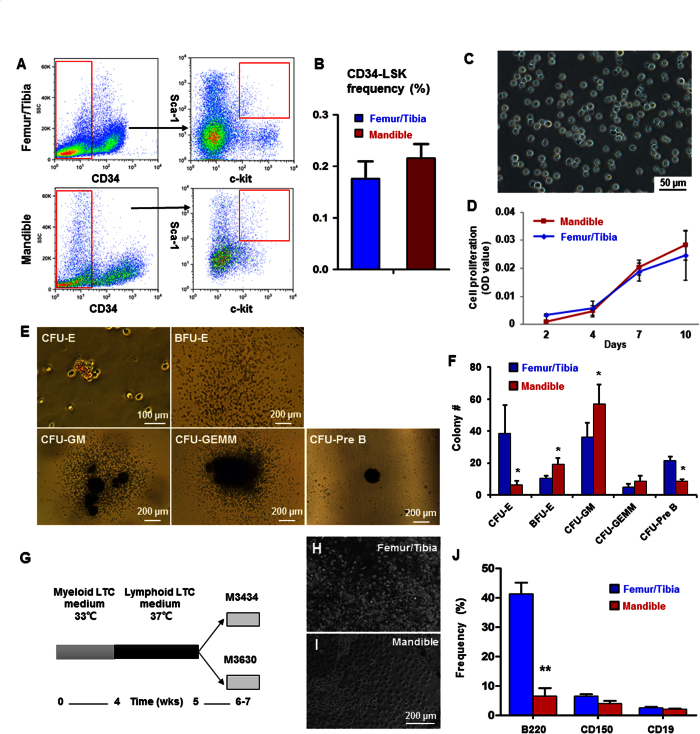
Characterization of HSC-like cells in neural-crest derived mandible. (**A**) Flow cytometry profiles of CD34ˉLSK cells. Lineage-depleted cells with CD34 negative and c-Kit and Sca-1 double positive selection. (**B**) Quantitative counts of CD34ˉLSK cells from femur/tibia and mandible at 0.176 ± 0.034 and 0.216 ± 0.027, respectively (mean ± s.d., n = 5). (**C**) Sorted mandibular CD34ˉLSK cells cultured for 10 days. (**D**) Proliferation rates of CD34ˉLSK cells from mandible and femur/tibia (mean ± s.d., n = 3). (**E**) Representative images of colony-forming mandibular CD34ˉLSK cells, including colony-forming unit erythroid (CFU-E), mature burst-forming unit-erythroid (BFU-E), colony-forming unit granulocyte/macrophage (CFU-GM), CFU-granulocyte/ erythroid/ megakaryocyte/ macrophage (CFU-GEMM) and pre-B lymphoid (CFU-Pre B). (**F**) Colony numbers of mandibular CD34ˉLSK cells (mean ± s.d., n = 3, *p < 0.05). (**G**) Diagram of LTC-IC assay. (**H,I**) HSCs cultured on a feeder layer in the myeloid medium for 4 wks and lymphoid medium for 1 wk. (**J**) Flow cytometry of differentiated long-term culture-initiating cells in methylcellulose culture medium (mean ± s.d., n = 3, **p < 0.01).

The mandibles of 8-wk-old C57/B6 mice were dissected to isolate total mononucleated cells following removal of all the teeth and the mandibular condyles. Bone marrow cells from the mandibular and femur/tibia bones were negatively selected by magnetic beads with lineage markers, followed by CD34-/low selection and Sca-1 and c-kit double-positive selection per prior protocols^25^,^26^. Total CD34 negative, LSK cells in the mandible accounted for 0.216 ± 0.027% of all total mononucleated cells, with no statistically significant differences from total CD34 negative, LSK cells in the femoral/tibial bones at 0.176 ± 0.034 (Fig. 1A,B). Sorted CD34ˉLSK cells were cultured in serum-free medium for 10 days and showed no attachment to culture plate (Fig. 1C), likely free from mesenchymal cell contamination. Mandibular and femoral/tibial CD34ˉLSK cells showed virtually the same proliferation rates (Fig. 1D). Mandibular CD34ˉLSK cells differentiated into colony-forming unit erythroid (CFU-E), mature burst-forming unit-erythroid (BFU-E), colony-forming unit-granulocyte/ macrophage (CFU-GM), CFU-granulocyte/erythroid/megakaryocyte/macrophage (CFU-GEMM) and pre-B lymphoid (CFU-Pre B) progenitors (Fig. 1E). Mandibular CD34ˉLSK cells showed robust potential to differentiate to myeloid lineages, but a deficiency towards the Pre-B lymphoid lineage in comparison to donor-matched femur/tibia CD34ˉLSK cells (Fig. 1F). Cells expressing myeloid markers such as TER119, CD14, CD41 and F4/80 were significantly more abundant in mandibular cells than donor-matched tibia/femur cells (Supplementary Fig. S1A,B). Contrastingly, cells expressing B-lineage markers including B220 and CD19 were significantly more abundant in mandibular Pre-B colonies than donor-matched tibia/femur samples (Supplementary Fig. S1C,D).

### LTC-IC indicated a lineage bias of mandibular HSCs

Assay for long-term culture-initiating cells (LTC-IC) allows a subset of LTC-IC to show their myeloid and lymphoid differentiation potential based on their capacity to produce progenies^27^. Freshly isolated femoral/tibial marrow cells were cultured in 24-well plates for 1 wk and subjected to irradiation at 1500 cGy to prevent cell confluence (data not shown). Hematopoietic cells isolated freshly from bone marrow of femur/tibia and the mandible were cultured on the feeder layer for 4 wks at 33 °C in myeloid medium, followed by 1-wk culture in lymphoid medium (RPMI1640 medium) with supplements at 37 °C (Fig. 1G), and seeding in the methylcellulose culture medium and tested by colony-forming cell assay (Fig. 1H,I). No significant differences in myeloid progenies markers were detected between mandibular and femoral/tibial marrow HSCs (Supplementary Fig. S1E). In the lymphoid progeny, however, cells expressing CD45R/B220 was ~20% less abundant in the mandible than donor-matched femur/tibial marrow HSCs (Fig. 1J), consistent with short-term assay above (Fig. 1A–F), in demonstrating that mandible HSCs have a lymphoid differentiation defect.

### SLAM markers showed deficient CD229 positive cells in the mandible

**Figure 2:**
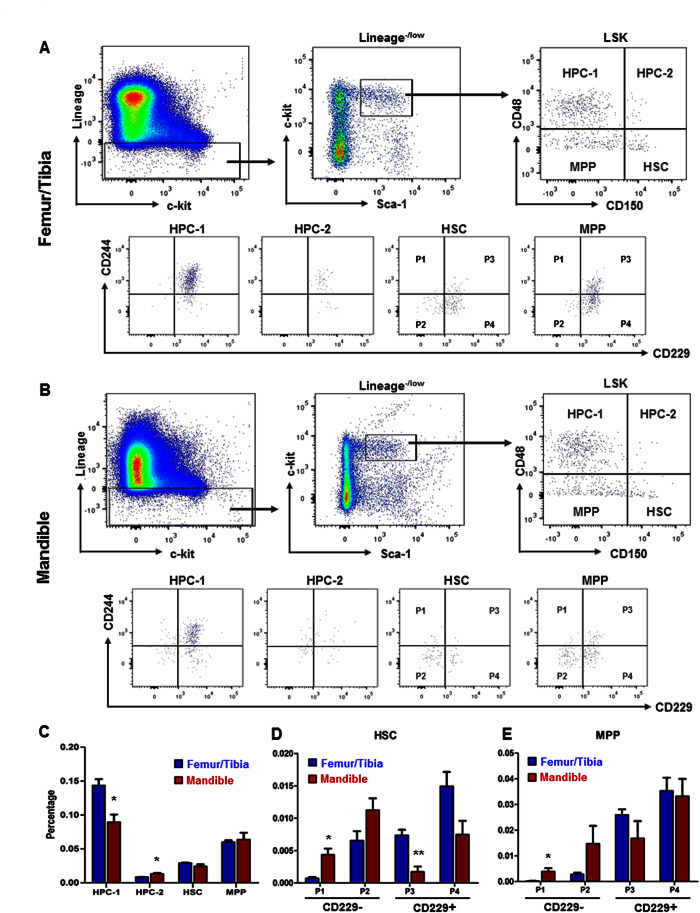
SLAM family markers by LSK HSC stem/progenitors. (**A,B**) Gating for femur/tibia and mandibular LSK cells, CD150 and CD48. The CD150^−^CD48^+^ LSK was labeled as HPC-1, CD150^+^ CD48^+^ LSK were labeled as HPC-2, CD150^+^ CD48^−/low^ LSK were labeled as HSC and CD150^−^CD48^−/low^ LSK were labeled as MPP. Doublets, red blood cells, and dead cells were excluded prior to analysis. HSC and MPP were gated by CD229 and CD244. CD229^−^CD244^+^. CD229^−^CD244^−^, CD229^+^ CD244^+^ and CD229^+^ CD244^−^ HSC/MPP were labeled as P1, P2, P3 and P4, respectively. (**C**) Frequency subdivided by SLAM markers (mean ± s.d., n = 3, *p < 0.05). (**D,E**) Frequency of stem and progenitor fractions subdivided by CD229 and CD244 of HSC and MPP populations. (mean ± s.d., n = 3, *p < 0.05, **p < 0.01).

SLAM family receptors distinguish subpopulations of HSCs and multipotent progenitors^28^,^29^. Accordingly, we tested marrow cells from femoral/tibial and mandibular bones with SLAM family markers (Fig. 2A,B). Mandibular and femur/tibia HSCs showed similar SLAM (including CD150, CD48, CD229 and CD244) frequency (Fig. 2A,B). Significantly fewer HPC-1 (CD150^−^CD48^+^ LSK) cells were detected in the mandible (0.090 ± 0.011) than femur/tibia (0.144 ± 0.009) (Fig. 2C), whereas significantly more HPC-2 (CD150^+^ CD48^+^) LSK cells were identified in the mandible (0.013 ± 0.001) (Fig. 2C). Mandibular HSCs contained significantly more CD229^−^ (P1 and P2) subpopulation and significantly less CD229^+^ population (Fig. 2D), with a similar trend for the MPP population (Fig. 2E), suggesting a lineage bias of the lymphoid in both HSC and MPP populations.

### B-lineage progenitors are deficient in the mandible

**Figure 3:**
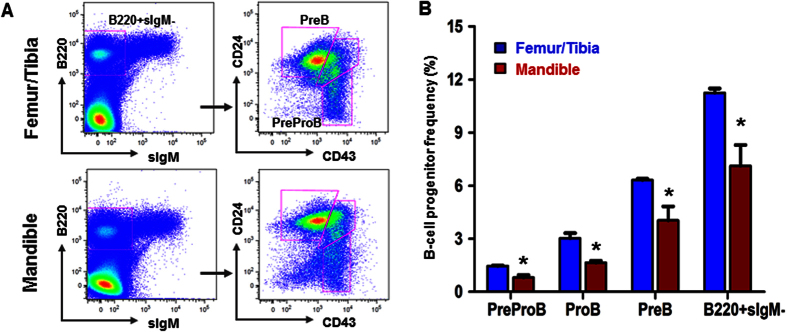
Flow cytometry for isolation of lymphoid progenitor cells. (**A**) Representative gates to isolate lymphoid progenitor cells. B220 + sIgM-CD43 + CD24- labeled as Pre-Pro B cells; B220 + sIgM-CD43 + CD24 + labeled as Pro B cells; B220 + sIgM-CD43- labeled as Pre B cells. (**B**) Frequency of lymphoid progenitor cells. (mean ± s.d., n = 3, *p < 0.05).

B-cell precursors are classified into four populations according to their differential expression of multiple cell-surface markers during development in bone marrow^30^. Here, we tested the frequency of B-lineage progenitor markers of mandibular and femur/tibia marrow cells. B-lineage progenitors from different locations were defined and scaled in Fig 3A. HSCs from femur/tibia showed significantly greater capacity to differentiate to B-lineage progenitors than mandibular HSCs (Fig. 3B). The decrease of B-lineage progenitors confirmed lineage-biased hematopoietic stem cells and progenitors in neural-crest derived mandibles.

### Hematopoietic recovery following bone-marrow transplantation

**Figure 4:**
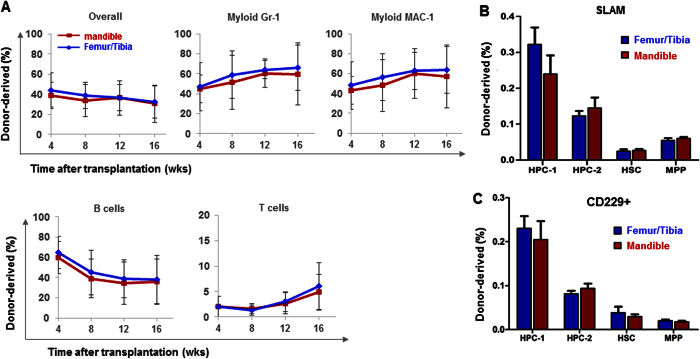
*In vivo* rescue of irradiated bone marrow. (**A**) A total of 3 × 10^5^ donor HSC-like cells from the mandible and from femur/tibia both reconstituted marrow hematopoiesis following sublethal irradiation, with similarly restored levels of myeloid cell, B-cell and T-cell (mean ± s.d., three experiments with a total of 12–15 recipient mice per group). (**B**) Frequency of stem and progenitor cells subpopulated by SLAM family markers (mean ± s.d., n = 5). (**C**) Frequency of CD229 positive cells of stem and progenitor cells (mean ± s.d., n = 5).

To test whether the lineage bias was contributed by local HSCs or influenced by neural-crest bone marrow niche, we then compared the reconstitution ability of femoral/tibial and mandibular hematopoietic stem/progenitor cells following bone-marrow transplantation. Donor cells were isolated and transplanted into irradiated recipient mice along with same number recipient bone marrow cells. Neural crest-derived mandibular HSCs gave rise to all major hematopoietic lineages in reconstituted bone marrow of irradiated mice including myeloid (Gr-1 + and MAC-1 + ), B cells and T cells, similar to femur/tibia HSCs (Fig. 4A). Mandibular HSCs benchmarked with SLAM and CD229 markers with femur/tibia HSCs including HPC1, HPC-2, HSC and MPP (Fig. 4B,C), suggesting that mandibular HSCs are as capable of restoring irradiated bone marrow as donor-matched femur/tibia HSCs.

### Gene profiling of HSC niche signals in femur/tibia and mandible

**Figure 5:**
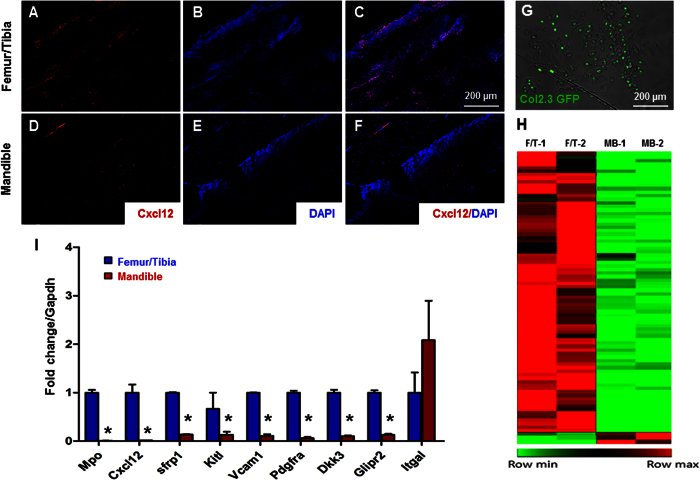
Profiling of HSC niche signals. (**A–C**) Cxcl12 immunofluorescence of the femur/tibia and (**D–F**) mandible. (**G**) Representative images of GFP positive, osteoblasts isolated from the mandible. (**H**) Heat map of global gene expression with significant change (p < 0.05). Up-regulated genes are indicated as red color and down-regulated genes are indicated as green color. (**I**) Selected genes differentially expressed in femur/tibia and mandible by using qRT-PCR (mean ± s.d., n = 3, *p < 0.05).

Given the observed similarities and differences between femur/tibia and mandibular HSCs, we analyzed the expression of Cxcl12, a significant chemokine in HSCs niches and regulating lymphoid differentiation. Cxcl12 expression was confirmed by immunofluorescence staining in bone marrow of both femur/tibia (Fig. 5A–C) and mandible samples (Fig. 5D–F). Less Cxcl12 were detected in the mandibular bone, indicating lymphoid deficiency may be mediated by insufficient Cxcl12. Then we profiled femur/tibia and mandibular osteoblasts to understand HSC niche cells. In Col-2.3 GFP mice, GFP expression is restricted to osteoblasts when cells begin to express collagen 1α1 chain (Fig. 5G)^31^,^32^. Col-2.3 GFP positive osteoblasts were isolated from femur/tibia and mandibular bone marrow by flow cytometry. Total RNA was extracted from GFP positive cells for RNA-seq (Fig. 5H). Specifically, several niche regulators were significantly down-regulated, such as Cxcl12, in mandibular osteoblasts compared with femur/tibia osteoblasts, which was confirmed by qRT-PCR (Fig. 5I). We also analyzed the biological processes enriched in genes that changed most significantly by using Ingenuity Pathway Analysis (IPA). Analysis revealed that several enriched pathways were up-regulated in favor of tibia/femoral osteoblasts (Supplementary Fig. S2), including IL-8, STAT3, EGF, and PDGF signaling. Thus, our data suggested that mandibular niche cells and signal molecules chemokine may contribute to the neural-crest HSCs lymphoid lineage bias.

**Figure 6:**
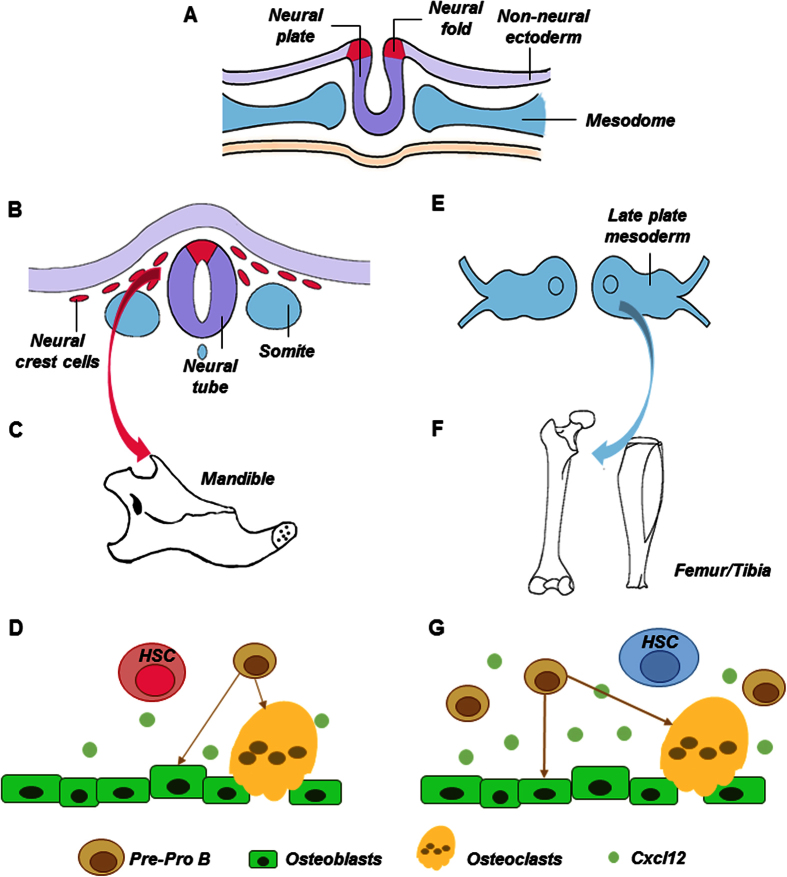
Schematics of neural-crest derived vs. mesoderm derived hematopoietic stem cells. Neural tube derives from neural epithelium early in development, with adjacent paraxial mesoderm (**A**). Neural crest cells split from neural tube and begin to migrate towards the presumptive face and form the first branchial arch (**B**), from which the mandible is derived (**C**). Here, we demonstrated that neural-crest derived bone (mandible) harbors HSCs that benchmark with mesoderm derived femur/tibia HSCs, and yet with a severe deficiency in lymphoid differentiation (**D**). Signals in HSC niche in the form of bound or secreted molecules, including osteoblast derived Cxcl12, regulate HSC functions (**D**). Contrastingly, HSCs derive from paraxial mesoderm reside in appendicular bones such as femur and tibia (**E**,**F**) and have been well characterized, including progenitors of osteoclasts and Cxcl12 as a pivotal signal that regulates HSC niche (**G**).

A schematic of neural crest-derived vs. mesoderm-derived hematopoietic stem cells is provided as Fig. 6. Neural plate (neural ectoderm) border cells bend to form the neural folds early in development, with adjacent paraxial mesoderm (Fig. 6A). Neural plate border cells eventually become the dorsal part of neural tube. Neural crest cells split from the dorsal neural tube and begin to migrate towards the presumptive face and form the first branchial arch (Fig. 6B), from which the mandible is derived (Fig. 6C). Here, we demonstrated that neural crest-derived bone (mandible) harbors HSCs that benchmark with mesoderm derived femur/tibia HSCs (Fig. 6E), and yet with a deficiency in the lymphoid lineage. Signals in HSC niche in the form of bound or secreted molecules, including deficient osteoblast-derived Cxcl12, may regulate mandibular lymphoid progenitor functions (Fig. 6D). Contrastingly, HSCs derived from paraxial mesoderm reside in appendicular bones such as femur and tibia (Fig. 6F), including pre-osteoclasts and Cxcl12 as a pivotal signal that regulates HSC niche (Fig. 6G).

## Discussion

The present findings suggest that neural crest-derived mandible harbors HSCs. HSCs in neural crest-derived bone marrow replenish all hematopoietic lineages, and are able to restore irradiated bone marrow, just as donor-matched mesoderm-derived appendicular HSCs. Postnatal HSCs in the jaw bone corroborate by robust hematopoiesis with long-term, self-renewable HSCs in mouse embryonic head at E12.5^12^. It is conceivable that subsets of the abundant HSCs at E12.5 maintain their stemness and self-renew ability and remain as postnatal HSCs that we detected here. Previously, postnatal stem/progenitor cells in craniofacial regions including the oral cavity have been identified entirely from the mesenchymal compartment, including mesenchymal stem cell (MSC)-like cells in dental pulp, periodontal ligament, apical papilla, oral mucosa and alveolar bone^13^. The present findings provide the basis for additional studies on the interactions of HSCs with dental craniofacial MSCs in the context of not only homeostasis including osteoclastogenesis, but also a multitude of pathological conditions including fracture healing, osteonecrosis, bone infections and osteonecrosis. Clearly, much is to be understood regarding craniofacial HSCs including their roles in homeostasis and perhaps pathological conditions such as osteonecrosis of the jaw.

Mandibular CD34ˉLSK cells not only differentiated into all hematopoietic lineages per CFU assays, but also showed strong potential to the myeloid lineage, comparable to donor-matched tibia/femur CD34ˉLSK cells. In the LTC-IC assay, the myeloid lineage differentiation potential of mandibular HSCs was similar to that of femur/tibia HSCs. We further distinguished subsets of hematopoietic stem/progenitor cells with several SLAM family markers. Lineage^−/low^Sca-1^+^ c-kit^+^ (LSK) cells are subdivided into MPPs (CD150^−^CD48^−^ LSK), HPC (CD48^+^ LSK) and HSCs (CD150^+^ CD48^−^ LSK)^28^. Besides CD150 (Slamf1) and CD48 (Slamf2), two other SLAM family markers, CD229 (Slamf3) and CD244 (Slamf4) further divide mouse bone marrow LSK cells into seven functionally subpopulations^29^. Mandibular marrow showed a significant deficiency of HPC-1 (CD150-CD48 + LSK) and a concomitant increase of HPC-2 (CD150 + CD48 + LSK). HPC-1 cells are a heterogeneous population of restricted progenitors including early lymphoid progenitors and those with low levels of transient myeloerythroid reconstitution. HPC-2 cells represent a heterogeneous cluster of restricted progenitors with limited reconstituting potential^29^. Probing of sub-HSC populations with CD244 and CD229, CD229 expression distinguished lymphoid-biased HSCs from rarely dividing myeloid-biased HSCs. Deficiency of mandibular CD229 + cells not only in HSCs, but also MPPs suggests a lineage bias of the lymphoid progenitors and MMPs. A complex array of signals mediate HSC maintenance. Several homeobox genes have been implicated in both branchial arch and limb development, including Dlx, Msx and Lim-homeobox families^33^,^34^,^35^. FGFs have different expression patterns between calvarial and limb development^36^. Whether a lymphoid deficiency of the mandibular HSCs is attributed by their neural crest origin or a postnatal functional needs additional investigations. Osteonecrosis of the jaw (ONJ) is a pathological condition frequently caused by bisphosphonate administration in organ transplant patients and represents dysregulated and destructive bone breakdown^37^. ONJ is virtually restricted to orofacial bones, and seldom affects appendicular skeleton, which yet remains an enigma^38^. The most popular hypothesis is bone remolding suppression, resulting from reduction of osteoclast-mediated bone remodeling and decrease of osteoblast-mediated systemic bone formation^39^,^40^. Both osteoblasts and osteoclasts were found to have direct cell-cell contact with lymphocytes^41^. There is evidence that bone homeostasis and bone mineral density will change when B cell differentiation is altered^42^. The loss of RANKL-producing B cells was found to affect osteoclast development and differentiation^43^. B lymphocytes also affects G-CSF induced hematopoietic stem/progenitor cells mobilization and osteoblast reduction^44^. Taken together, the presence of HSCs with lymphoid deficiency in craniofacial bones may shed some light on the mechanisms and novel therapeutics of ONJ.

Hematopoietic stem cell niche maintains and regulates HSCs homeostasis and regenerative potential, which consists of cells and signals. Among multiple niche-critical genes, Cxcl12 is an indispensable factor regulating HSCs retention, quiescence and multilineage reconstitution^16^. To interpret neural-crest bone marrow hematopoietic lineage bias, we first tested the Cxcl12 in femur/tibia and mandible bone marrow and found an obvious decrease expression in the neural-crest derived bone. HSCs are known to interact with niche cells including stromal cells and osteoblasts in the endosteum of bone marrow^45^,^46^. Evidence showed that osteoblast lineage are dispensable for HSC maintenance but may regulate lymphoid progenitors^19^,^23^. We then compared donor-matched osteoblasts from the mandible and appendicular bones in Col2.3 GFP mouse by RNA-seq. Among dozens of decreased genes, Cxcl12′s significant deficiency in mandibular osteoblasts may have broad implications in HSC niche. Cxcl12 is required for the maintenance of certain early lymphoid progenitors but not the maintenance of HSCs and cellularity^23^,^47^. The depletion of early lymphoid progenitors could be restored by downstream progenitors in mesoderm-derived femur/tibia but not in the neural-crest derived bones. Other niche factors regulating HSC function was also down-regulated, per our RNA-seq data and Ingenuity Pathway Analysis. For example, interleukin-8 signaling induces neutrophils that are indispensable for hematopoietic stem cell mobilization^48^. STAT3 signaling is another key factor regulating HSCs self-renew during hematopoietic repopulating activities^49^. PDGFRα as a marker for niche capability of hematopoietic progenitor cell expansion^50^ also is deficient in the mandible. Neural crest-derived stromal cells and signals in facial bones preserve different stromal microenvironment for HSCs, which may contribute to not only lymphoid but myeloid as well. Facial bones including the maxilla and mandible have rich vasculature^51^, with bone marrow stromal cells proliferating at more rapid rates, with delayed senescence. These speculations warrant additional investigations to understand HSC niche in neural crest derived bones.

## Methods

All methods were carried out in accordance with relevant guidelines and regulations.

### Mice

Following IACUC approval, 8-wk-old C57/B6 mice were sacrificed with total bone-marrow cells isolated from femurs and tibiae, as well as the mandibles. Col2.3-GFP mice (Jackson Laboratories) expressed enhanced green fluorescent protein gene under the control of 2.3 kb mouse procollagen, type 1, alpha 1(Col1a1) promoter. All experimental protocols were approved by Columbia University IACUC committee.

### Flow cytometry

8-wk-old C57/B6 mice were dissected to isolate total femur/tibia and mandibular bone (with removal of all the teeth and the mandibular condyles) mononucleated cells by crushing with a mortar and pestle in Ca2 + and Mg2 + free HBSS with 2% heat-inactivated bovine serum. Cells were dissociated to single cell suspension by filtering through a 70-mm nylon mesh. For CD34ˉLSK cells isolation, lineage + cells were excluded by magnet selection^26^ with a mouse hematopoietic progenitor cell isolation kit (Stemcell Technology, Vancouver, BC, Canada). Anti-CD34 (RAM34), anti-Sca1 (E13-161.7), anti-c-KIT (2B8) antibodies were used to isolate CD34^−/low^c-Kit^+^ Sca-1^+^ lineage marker (CD34ˉLSK) cells. The following antibodies were used to further characterize the cells: anti-Ter119/Ly-76 (TER-119), anti-CD71 (R17217), CD41 (MW Reg30), CD14 (rmC5-3), F4/80 (BM8), anti-GR1 (8C5), CD11b (M1/70), anti-B220/CD45R (RA3-6B2), CD19 (1D3) and CD150 (TC15-12F12.2). The following HSCs antibodies were used as lineage markers: anti-CD2 (RM2-5), anti-CD3 (17A2), anti-CD5 (53-7.3), anti-CD8 (53-6.7), anti-TER119, anti-B220, anti-GR1, anti-CD150 (TC15-12F12.2), anti-CD48 (HM48-1), anti-CD229 (Ly9ab3) and CD244.2 (2B4). Hematopoietic PreProB progenitors were characterized with the following antibodies: anti-B220, anti-IgM (II/41), anti-CD43 (1B11) and anti-CD24 (M1/69). DAPI was used to exclude dead cells.

### Short time culture

Sorted CD34ˉLSK cells were cultured in StemSpan SFEM medium (Stem Cell Technology) suspended with purified recombinant mouse SCF (10 ng/ml, R&D Systems, Minneapolis, MN), human TPO (10 ng/ml, R&D Systems), human Flt-3L (10 ng/ml, R&D Systems) and mouse IL-3(10 ng/ml, R&D Systems)^52^,^53^,^54^. Cell proliferation was measured at 490 nm using CellTiter 96 AQueous One Solution Cell Proliferation Assay (Promega, Madison, WI).

### Colony-forming assay

Colony-forming assay was performed per prior methods^55^. Briefly, 1 × 10^3^ cells were cultured in methylcellulose culture medium (M3334, M3434 and M3630, Stemcell Technology) with recombinant cytokines and incubated at 37 °C as instructed. Colony-forming units in culture were subsequently scored under inverted microscope. Colony-forming unit Erythroid (CFU-E), mature burst-forming unit-erythroid (BFU-E), colony-forming unit (CFU)-granulocyte/macrophage (GM), CFU-granulocyte/erythroid/megakaryocyte/macrophage (GEMM) and pre-B lymphoid (CFU-Pre B) progenitor numbers were defined as previous studies. Then cells were trypsinized and further stained to perform flow cytometry.

### Long-Term Culture-Initiating Cell (LTC-IC)

Bone-marrow cells for feeder layer were flushed by using a 21-gauge needle into cold α-MEM medium (Invitrogen, Carlsbad, CA) with 2% fetal calf serum. Single-cell suspension was obtained by repeated gentle aspiration and plating. Cells were maintained in MyeloCult M5300 with 10^−3^ Hydrocortisone at 33 °C with medium change every 3 days and concomitant removal of half of the non-adherent cells. Upon 70–80% confluence, cells were irradiated at I500 cGy from a 137Cs r-irradiation source. Cells were then cultured onto the feeder layer and maintained in the same culture medium for 4 wks, followed by separately harvesting pooled adherent and non-adherent fractions for BFU-E, CFU-GM, and CFU-GEMM assays. For lymphoid differentiation, a two-stage culture system was used. Cells were initially seeded onto pre-established irradiated marrow feeders for 4 wks, followed by myeloid culture and removal of non-adherent cells. Adherent cell layer was washed twice with warm RPMI 1640 (Invitrogen) and subjected to RPMI1640 supplemented with 5% fetal calf serum and 50 μmol β-ME (Sigma-Aldrich, St. Louis, MO) at 37 °C for 1 wk. Trypsinized adherent cells were pooled with their corresponding non-adherent cells for lymphoid colony assay^27^,^56^.

### Long-term competitive reconstitution assay

Adult mice were given a minimum lethal dose of radiation using a Cesium 137 GammaCell 40 Irradiator (MDS) to deliver two doses of 540 rad (1,080 rad total) at least 2 hrs apart. Donor cells were injected into the retro-orbital venous sinus of anaesthetized C57BL/6-SJL (CD45.1) mice as the recipient. Mononucleated cells were counted before transplantation using a haemocytometer. Recipient mice were maintained on antibiotic water (1.11 gl^−1^ neomycin sulphate and 0.121 gl^−1^ polymixin B) for 14 days following transplantation and then switched to regular water. Recipient mice were bled from 4 to 16 wks following transplantation to examine donor-derived myeloid, B and T cells in their blood with the following antibodies to analyze donor chimaerism: anti-CD45.1 (A20), anti-CD45.2 (104), anti-GR1 (8C5), anti-Mac1 (M1/70), anti-B220 (6B2) and anti-CD3 (KT31.1).

### Immunofluorescence

Eight-wk-old C57/B6 mice were sacrificed with mandibles and femurs freshly dissected followed by 4%-formalin fixation. Tissue sections were incubated with anti-Cxcl12 antibody (1:400, Abcam, Cambridge, MA) overnight at 4 °C followed by secondary antibodies, Alexa Fluor® 647 Goat Anti-Rabbit IgG (H + L) Antibody (1:2000, Invitrogen).

### RNA sequencing and profiling

Total RNA was isolated per mRNA Isolation Kit (Invitrogen). The quality of total RNA was verified by an Agilent 2100 Bioanalyzer profile with RIN number >8.0. The extracted mRNAs were isolated and reverse transcribed into cDNA using the mRNA-Seq preparation kit (Illumina, San Diego, USA). RNA sequencing was performed at UR Genomics Research Center. Illumina Hiseq platform was used, generating 100bp paired end reads. Differentially expressed genes were selected for network construction abd uploaded into ingenuity pathway analysis (IPA) software. Quantitative RT-PCR (TaqMan) was performed to validate gene expression with targeted mRNA expression normalized to GAPDH.

### Data analysis and statistics

Upon confirmation of normal data distribution, all quantitative data of control and treated groups were treated with two-tailed t-tests with an α level of 0.05. All experiments were repeated at least three times.

## Additional Information

**How to cite this article**: Jiang, N. *et al*. Hematopoietic Stem Cells in Neural-crest Derived Bone Marrow. *Sci. Rep.* **6**, 36411; doi: 10.1038/srep36411 (2016).

**Publisher's note:** Springer Nature remains neutral with regard to jurisdictional claims in published maps and institutional affiliations.

## Supplementary Material

Supplementary Information
