# Supplementary material for: Hematopoietic Stem Cells in Neural-crest Derived Bone Marrow
Source: Sci Rep. 2016 Dec 21;6:36411. doi: 10.1038/srep36411 (PMC5175267; doi:10.1038/srep36411)

Supplementary Information

Hematopoietic Stem Cells in Neural-crest Derived Bone Marrow

Nan Jiang[1,2,3], Mo Chen[2], Guodong Yang[2], Lusai Xiang[2,4], Ling He[2,4], Thomas K. Hei[5],
Gregory Chotkowski[2], Dennis P. Tarnow[6], Myron Finkel[2],
Lei Ding[7], Yanheng Zhou[1]*, Jeremy J. Mao[2,4]*

[1]Department of Orthodontics, Peking University School and Hospital of Stomatology
22 Zhongguancun Avenue South, Haidian District, Beijing 100081, P.R. China
[2]Columbia University Medical Center, Center for Craniofacial Regeneration (CCR)
630 W. 168 St., New York, NY 10032, USA
[3]Central Laboratory, Peking University School and Hospital of Stomatology,
22 Zhongguancun Avenue South, Haidian District, Beijing 100081, China
[4]Guanghua School of Stomatology, Hospital of Stomatology, Sun Yat-sen University
56 Lingyuanxi Road, Guangzhou 510055, China
[5]Department of Radiation Oncology, College of Physician and Surgeons
Columbia University Medical Center, 630 W. 168 St., New York, NY 10032, USA
[6]Division of Periodontics, College of Dental Medicine, Columbia University Medical Center
630 W. 168 St., New York, NY 10032, USA
[7]Departments of Microbiology & Immunology and Rehabilitation & Regenerative Medicine
630 W. 168 St., New York, NY 10032, USA

Supplementary Figure S1. Flow cytometry profiles were shown for analysis of CD34⁻LSK cells differentiation in M3434 and M3630. (A) Flow cytometry of differentiated colonies for myeloid markers. (B) Significant increases of Ter119, CD14, CD41 and F4/80 (mean ± s.d., n=3, *p<0.05, **p<0.01, t-tests). (C) Flow cytometry of differentiated lymphoid colonies. (D) Significant decrease of B220 and CD19 (mean ± s.d., n=3, *p<0.05, **p<0.01, t-tests). (E) Flow cytometry for analysis of LTC-IC myeloid differentiation in M3434. (mean ± s.d., n=3, t-tests).

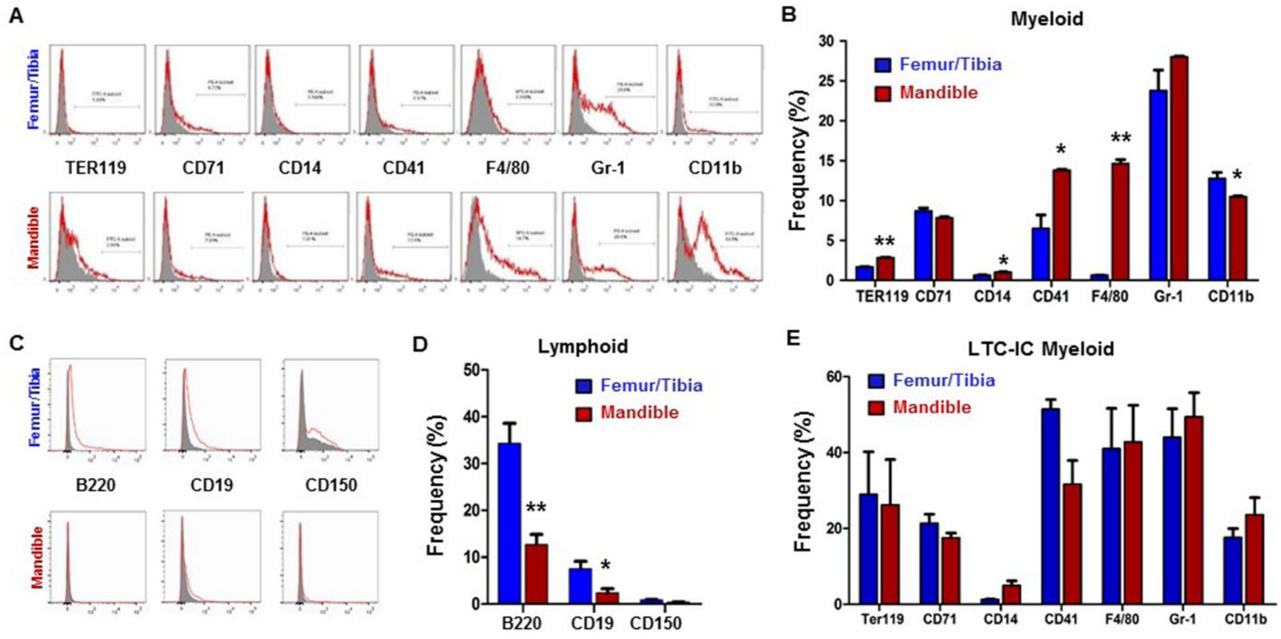

Supplementary Figure S2. Selected enriched pathways femur/tibia vs. mandible with statistical significance. The – log (P value) of the enrichment of each canonical pathway was plotted.

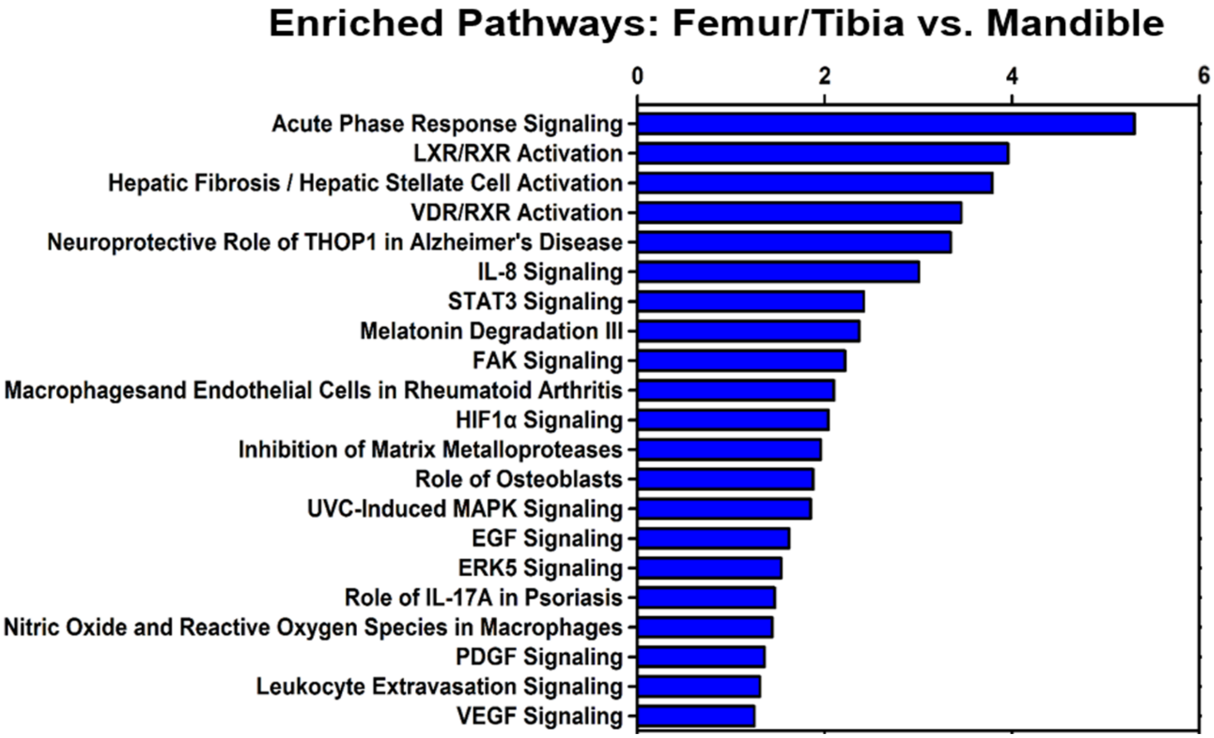

Supplement: Supplementary Information [file srep36411-s1.pdf]
